# Multi-cohort modeling strategies for scalable globally accessible prostate cancer risk tools

**DOI:** 10.1186/s12874-019-0839-0

**Published:** 2019-10-15

**Authors:** Johanna Tolksdorf, Michael W. Kattan, Stephen A. Boorjian, Stephen J. Freedland, Karim Saba, Cedric Poyet, Lourdes Guerrios, Amanda De Hoedt, Michael A. Liss, Robin J. Leach, Javier Hernandez, Emily Vertosick, Andrew J. Vickers, Donna P. Ankerst

**Affiliations:** 1 Departments of Mathematics and Life Sciences, Technical University of Munich, Boltzmannstr.3, 85747 Garching near Munich, Germany; 2 Department of Quantitative Health Sciences, Cleveland Clinic, 9500 Euclid Avenue, Cleveland, OH 44195 USA; 3 Department of Urology, Mayo Clinic, 200 1st St SW W4, Rochester, MN 55905 USA; 4 Department of Urology, Durham Veterans Administration Medical Center, 508 Fulton St, Durham, NC 27705 USA; 5 Department of Surgery, Cedars-Sinai Medical Center, 8700 Beverly Blvd, Los Angeles, CA 90048 USA; 6 Department of Urology, University Hospital Zurich, University of Zurich, Rämistrasse 71, CH-8006 Zurich, Switzerland; 7 Department of Surgery, Urology Section, Veterans Affairs Caribbean Healthcare System, 10 Calle Casia, San Juan, 00921-3201 Puerto Rico; 8 Department of Urology, University of Texas Health Science Center at San Antonio, 7703 Floyd Curl Dr, San Antonio, TX 78229 USA; 9 Department of Epidemiology & Biostatistics, Memorial Sloan Kettering Cancer Center, 1275 York Avenue, New York, NY 10065 USA

**Keywords:** Risk prediction, Prostate cancer, Validation, Calibration, Discrimination, Net benefit

## Abstract

**Background:**

Online clinical risk prediction tools built on data from multiple cohorts are increasingly being utilized for contemporary doctor-patient decision-making and validation. This report outlines a comprehensive data science strategy for building such tools with application to the Prostate Biopsy Collaborative Group prostate cancer risk prediction tool.

**Methods:**

We created models for high-grade prostate cancer risk using six established risk factors. The data comprised 8492 prostate biopsies collected from ten institutions, 2 in Europe and 8 across North America. We calculated area under the receiver operating characteristic curve (AUC) for discrimination, the Hosmer-Lemeshow test statistic (HLS) for calibration and the clinical net benefit at risk threshold 15%. We implemented several internal cross-validation schemes to assess the influence of modeling method and individual cohort on validation performance.

**Results:**

High-grade disease prevalence ranged from 18% in Zurich (1863 biopsies) to 39% in UT Health San Antonio (899 biopsies). Visualization revealed outliers in terms of risk factors, including San Juan VA (51% abnormal digital rectal exam), Durham VA (63% African American), and Zurich (2.8% family history). Exclusion of any cohort did not significantly affect the AUC or HLS, nor did the choice of prediction model (pooled, random-effects, meta-analysis). Excluding the lowest-prevalence Zurich cohort from training sets did not statistically significantly change the validation metrics for any of the individual cohorts, except for Sunnybrook, where the effect on the AUC was minimal. Therefore the final multivariable logistic model was built by pooling the data from all cohorts using logistic regression. Higher prostate-specific antigen and age, abnormal digital rectal exam, African ancestry and a family history of prostate cancer increased risk of high-grade prostate cancer, while a history of a prior negative prostate biopsy decreased risk (all *p*-values < 0.004).

**Conclusions:**

We have outlined a multi-cohort model-building internal validation strategy for developing globally accessible and scalable risk prediction tools.

## Background

The widespread use of prostate-specific antigen (PSA) in combination with other established risk factors to assist clinicians in the early detection of prostate cancer has fostered user-friendly online risk tools for assessing the chance of prostate cancer detection if a prostate biopsy were to be performed. Patients and clinicians use risk estimates to assist their decision-making considering procession to biopsy. The first prostate cancer risk tools emanated in the mid-2000s following completion of large prostate cancer screening and prevention studies in Europe and North America [1, 2]. In addition to helping individual patients, their online accessibility enabled validation in heterogeneous populations ranging from similar to divergent from where they were developed [3, 4]. However, as technical, reporting and other changes occurred globally in prostate cancer, such as the systematic increase in the number of biopsy cores to increase detection, there came the need to collect contemporary real time data outside of the screening/prevention trial framework in order to expediently adapt the online risk tools to modern practice [5].

Towards this end the Prostate Biopsy Collaborative Group (PBCG) was formed to prospectively collect the standard risk factors and prostate biopsy outcomes from ten diverse international centers in Europe, North America and its territories [6]. The PBCG received funding to centralize data so that uniform analysis could be applied at the individual-patient level. However, a secondary aim of the PBCG was to find scalable methods for multi-cohort risk modeling that would enable the addition of cohorts into the future as well as the addition or modification of data from existing cohorts once funding for centralized data processing ceased.

In this report we provide the multi-cohort risk model development strategy used to develop the online PBCG prostate cancer risk tool [7]. In the spirit of data science, we propose informative visualization graphics for communication of cohort heterogeneity to partnering clinics. We identify five potential methods for performing logistic regression with clustered cohort data, including those working on the individual patient-level versus traditional meta-analysis methods that only work with study level aggregated summaries, commonly referred to in the literature as one- versus two-stage meta-analyses, respectively [8]. To compare modeling methods and assess the impact of individual cohorts on prediction we outline a comprehensive internal validation strategy for ensuring the globally intended tools are as accurate as possible for the widest berth of populations. Although demonstrated for a specific application in prostate cancer, we believe our comprehensive cohort visualization/model comparison/internal validation concept for creating globally intended risk tools generalizes to other clinical multi-cohort risk prediction applications intended for online use.

## Methods

### Patients and materials

We prospectively and retrospectively collected data from 10 PBCG sites under local ethics board approval at each of the sites. Participating sites included Cleveland Clinic, Mayo Clinic, San Raffaele, Zurich (University Hospital Zurich), MSKCC (Memorial Sloan Kettering Cancer Center), UCSF (University of California San Francisco), Durham VA (Veterans Affairs), VA Caribbean Health Care System (San Juan VA), Sunnybrook and UT Health (San Antonio) with data collected from 2006 to 2017. Biopsy results, including grade of prostate cancer, were collected along with the pre-biopsy risk factors PSA, digital rectal exam (DRE), age, African ancestry, first-degree family history of prostate cancer and whether or not a prior prostate biopsy that was negative for prostate cancer was ever performed. PSA was transformed to the log-base-2 scale (log2PSA) for improved fitting, and subsequently standardized by subtracting the mean and dividing by the standard deviation (across all sites) of the transformed values for inclusion in all analyses. Age was similarly standardized for all analyses. Description of the cohorts can be found in [7].

### Visualization of cohort differences and influence on risk factors

We created graphical displays using the ggplot2 package from the R statistical software to investigate heterogeneity among the 10 cohorts in terms of numbers of biopsies, prevalence of the high-grade (Gleason grade ≥ 7) prostate cancer outcome, distribution of risk factors, and odds ratios for association of the risk factors to high-grade disease [9, 10].

### Prediction models

The online PBCG risk tool built on these data and available at riskcalc.org used multinomial logistic regression for predicting the three outcomes of high-grade, low-grade, and no-cancer on biopsy based on the main effects log2PSA, age, DRE, African ancestry, family history and prior biopsy [7]. For this study, in anticipation of future updates to the online risk tool, we additionally included the interactions log2PSA:DRE, age:DRE, and age:African ancestry as they marginally improved the Bayesian Information Criterion (BIC), and performed logistic regression for predicting high-grade cancer versus the other outcomes low-grade and no cancer combined. We fixed the set of covariates for all subsequent analyses as feature selection was not the goal of this study. We completed the TRIPOD checklist for prediction model development [11].

With the fixed set of covariates, we compared five different methods for developing a prediction model, listed in Table 1. The first three methods pooled individual-level data from the cohorts and fit a single model. The first ignored the center effect altogether and the second two adjusted for it with a random effect. These methods required individual level data from the centers as opposed to the last two methods that used traditional meta-analysis, whereby only the coefficients and their corresponding variances from center-specific models were combined using fixed and random effects, respectively. Predictions from the five methods equaled the inverse logistic function, $\frac{\mathit{\exp}\left({\beta}_0+{\beta}^{\prime }x\right)}{\left\{1+\mathit{\exp}\left({\beta}_0+{\beta}^{\prime }x\right)\right\}},$ with *β*_0_ and *β* the coefficients from the respective fitting methods. We did not test whether or not random effects sufficiently improved goodness-of-fit in models that included them (Models 2, 3 and 5 of Table 1), but rather included the effects regardless in fitting the models. We then used the estimated fixed effects and considered two methods for handling predictions for new individuals based on random-effects models. The first set the random effect for the new individual to the prior mean value of 0 in the Normal distribution, termed median prediction by [12]. The second more commonly used method integrated the prediction, $\frac{\mathit{\exp}\left({\beta}_0+{\beta}_{0c}+{\beta}^{\prime }x\right)}{\left\{1+\mathit{\exp}\left({\beta}_0+{\beta}_{0c}+{\beta}^{\prime }x\right)\right\}},$ over the Normal distribution assumed for the random effects, *β*_0*c*_, using numerical integration in R [12].

**Table 1:** Five methods for optimal prediction in the validation; $logit\ x=\mathit{\log}\left\{\frac{x}{1-x}\right\}$, *y*_*i*_ = 1 high-grade cancer, 0 otherwise and *x*_*i*_ = vector of covariates for the *i* th individual for all individuals across all centers (*n* the total number of individuals), *β*_0_ a fixed intercept, *β* = (*β*_1_, …, *β*_*k*_, …, *β*_9_) a fixed vector of parameters of length 9 for the covariates log2PSA, age, DRE, African ancestry, family history and prior negative biopsy history, as well as the interactions log2PSA and DRE, age and DRE, age and African ancestry

Type of logistic regression	Model form	Risk predictor
1.Pooled data, cohort ignored	*logit P*(*y*_*i*_ = 1) = *β*_0_ + *β* ′ *x*_*i*_ by logistic regression fit to *i* = 1, …, *n* total number of patients	$\frac{\mathit{\exp}\left({\beta}_0+{\beta}^{\prime }x\right)}{\left\{1+\mathit{\exp}\left({\beta}_0+{\beta}^{\prime }x\right)\right\}}$
2.Pooled data, cohort as random effect, median prediction	*logit P*(*y*_*ic*_ = 1) = *β*_0_ + *β*_0*c*_ + *β*^′^*x*_*ic*_, *β*_0*c*_~*N*(0, *d*), by generalized linear mixed-effects models (binomial with logistic link) fit to *i* = 1, …, *n*_*c*_ patients in *c* = 1, …, *C* centers	$\frac{\mathit{\exp}\left({\beta}_0+{\beta}^{\prime }x\right)}{\left\{1+\mathit{\exp}\left({\beta}_0+{\beta}^{\prime }x\right)\right\}}$
3.Pooled data, cohort as random effect, mean prediction	*logit P*(*y*_*ic*_ = 1) = *β*_0_ + *β*_0*c*_ + *β*^′^*x*_*ic*_, *β*_0*c*_~*N*(0, *d*), by generalized linear mixed-effects models (binomial with logistic link) fit to *i* = 1, …, *n*_*c*_ patients in *c* = 1, …, *C* centers	${\int}_{-\infty}^{\infty}\frac{\mathit{\exp}\left({\beta}_0+{\beta}_{0c}+{\beta}^{\prime }x\right)}{\left\{1+\mathit{\exp}\left({\beta}_0+{\beta}_{0c}+{\beta}^{\prime }x\right)\right\}}f\left({\beta}_{0c}\right)d{\beta}_{0c},$ with *f*(*β*_0*c*_) density of *β*_0*c*_~*N*(0, *d*)
4.Meta-analysis, fixed effects by center	*logit P*(*y*_*i*_ = 1) = *β*_0_ + *β* ′ *x*_*i*_, with ${\beta}_k=\frac{\sum_{c=1}^C{w}_{kc}{\beta}_{kc}}{\sum_{c=1}^C{w}_{kc}},k=0,\dots, 9,$ *β*_*kc*_ estimated by separate logistic regressions for each center *c* = 1, …, *C*, *w*_*kc*_ = 1/ *var* (*β*_*kc*_), where *var*(*β*_*kc*_) is the within-center estimate of the variance of *β*_*kc*_.	$\frac{\mathit{\exp}\left({\beta}_0+{\beta}^{\prime }x\right)}{\left\{1+\mathit{\exp}\left({\beta}_0+{\beta}^{\prime }x\right)\right\}}$
5.Meta-analysis, random effects by center	*logit P*(*y*_*i*_ = 1) = *β*_0_ + *β* ′ *x*_*i*_, with ${\beta}_k=\frac{\sum_{c=1}^C{w}_{kc}{\beta}_{kc}}{\sum_{c=1}^C{w}_{kc}},k=0,\dots, 9,$ *β*_*kc*_ estimated by separate logistic regressions for each center *c* = 1, …, *C*, *w*_*kc*_ = 1/{*var*(*β*_*kc*_) + *b*}, where *var*(*β*_*kc*_) is the within-center estimate of the variance of *β*_*kc*_, and *b* the between-center estimate of variance based on a method-of-moments estimation.	$\frac{\mathit{\exp}\left({\beta}_0+{\beta}^{\prime }x\right)}{\left\{1+\mathit{\exp}\left({\beta}_0+{\beta}^{\prime }x\right)\right\}}$

### Missing data

Five biopsies from five patients were excluded due to missing age or Gleason score. For missing values in the four binary covariates, we imputed non-African, normal DRE, and no prior biopsy or family history. We investigated the impact of multiple imputation and found no impact on effect sizes or significance, apparently due to the large sample sizes and low percentage of missing data [13].

### Out-of-sample prediction criteria

We graded the performance of the risk tools in terms of discrimination, calibration, and net benefit. Metrics assessing these features are best observed as curves dependent on thresholds of the risk for referral to biopsy, as we have reported for the online PBCG risk tool [7]. Here due to the high number of internal validations performed, the curves are summarized in terms of relevant single number statistics, whose average and variation over different test sets can be computed.

For discrimination, we used the area under the receiver operating characteristic curve (AUC), which measures for a randomly chosen pair of persons with and without the clinical outcome of interest, the probability that the person with the outcome has a higher model-estimated risk [14]. The AUC ranges between 0.5, corresponding to random prediction, to 1.0, corresponding to perfect prediction.

For calibration, we used the Hosmer-Lemeshow test statistic (HLS) that summarizes differences between observed and expected counts according to risk decile groups [15]. Specifically, model-based predictions are calculated for all individuals of the test set, the individuals are sorted from lowest to highest risk, with the sample deciles of risk calculated to form the endpoints of ten intervals of risk, from the lowest 10% to the highest 10% of risk. Within each decile group, the average of the model-based risks is calculated as the expected risk and the sample proportion of individuals with the outcome as the observed risk. The HLS statistic is the sum of the squared difference between the observed – expected risks divided by the expected risks, and asymptotically follows a chi-square statistic with eight degrees of freedom. For the HLS, lower values as close to the minimal value of zero are better. To be comparable to the AUC, where higher values are better, we report the negative HLS (−HLS), where higher values also indicate better fit.

For net benefit we used the difference in clinical utility of referral to biopsy based on a model-computed risk compared to the strategy of referring all patients to biopsy [16]. For the cross-validation studies we used the net benefit at risk threshold 15% as it indicated net benefit for the online PBCG risk tool [7].

Both the AUC and HLS are single number summaries of curves across the risk prediction range, the latter of which provide more descriptive information as to which ranges of risk the predictions perform more poorly. We inspected these for the final model but not for the model selection process described here, because the permutation-based internal validation strategy summarized hundreds of different combinations of test sets. Calibration curves for the final model here are similar to those reported in [7].

### Internal validation

We compared the five modeling methods listed in Table 1 by computing differences in AUC, HLS and net benefit between each pair of methods over an internal validation over all 252 test sets that could arise from splitting ten cohorts into five used for training and five for testing. Similar to a prior multi-cohort study, we chose to split cross-validation by cohorts rather than by participants within cohorts because we were interested in the performance of a risk tool built on clustered data from heterogeneous centers that would be used by individual patients and/or validated in different centers [17]. However, repeated cross-validation at the individual level returned similar results. Performing the bootstrap, which samples cohorts with replacement instead of without replacement as the permutation strategy here does, also returned similar results. We favored the exhaustive approach of permuting over all possible sets of five cohorts that could be used for testing and training as this was the easiest manner to explain to partners how estimates and validation of a risk tool depend on the choice of cohorts selected to train the model as well as to test the model. We summarized the distribution of the validation metrics across all splits, referred to as the permutation distribution, by the median, 2.5 and 97.5% quantiles [18].

We investigated differences in AUCs and HLSs over the 252 test sets when all cohorts were included versus when each cohort was excluded to assess whether exclusion of the cohort resulted in improvement in prediction.

Zurich provided the largest cohort (1863 biopsies) that diverged the most from the rest in terms of lowest high-grade cancer prevalence (18% compared to 27% and higher in all other cohorts), no patients with African ancestry, lowest proportion of patients with positive family history (< 5%), and highest proportion with a prior negative biopsy (40%). To address whether Zurich should be included in the model-building cohorts, using each cohort sequentially as a sole test set, we inspected the difference in AUC, HLS and net benefit at 15% threshold between using all other cohorts as a training set versus all others excluding Zurich as a training set. 95% confidence intervals for the AUC were computed analytically, while the bootstrap was used to calculate 2.5 to 97.5 percentile intervals for the HLS and net benefit.

## Results

### Data summary and visualization

**Fig. 1:**
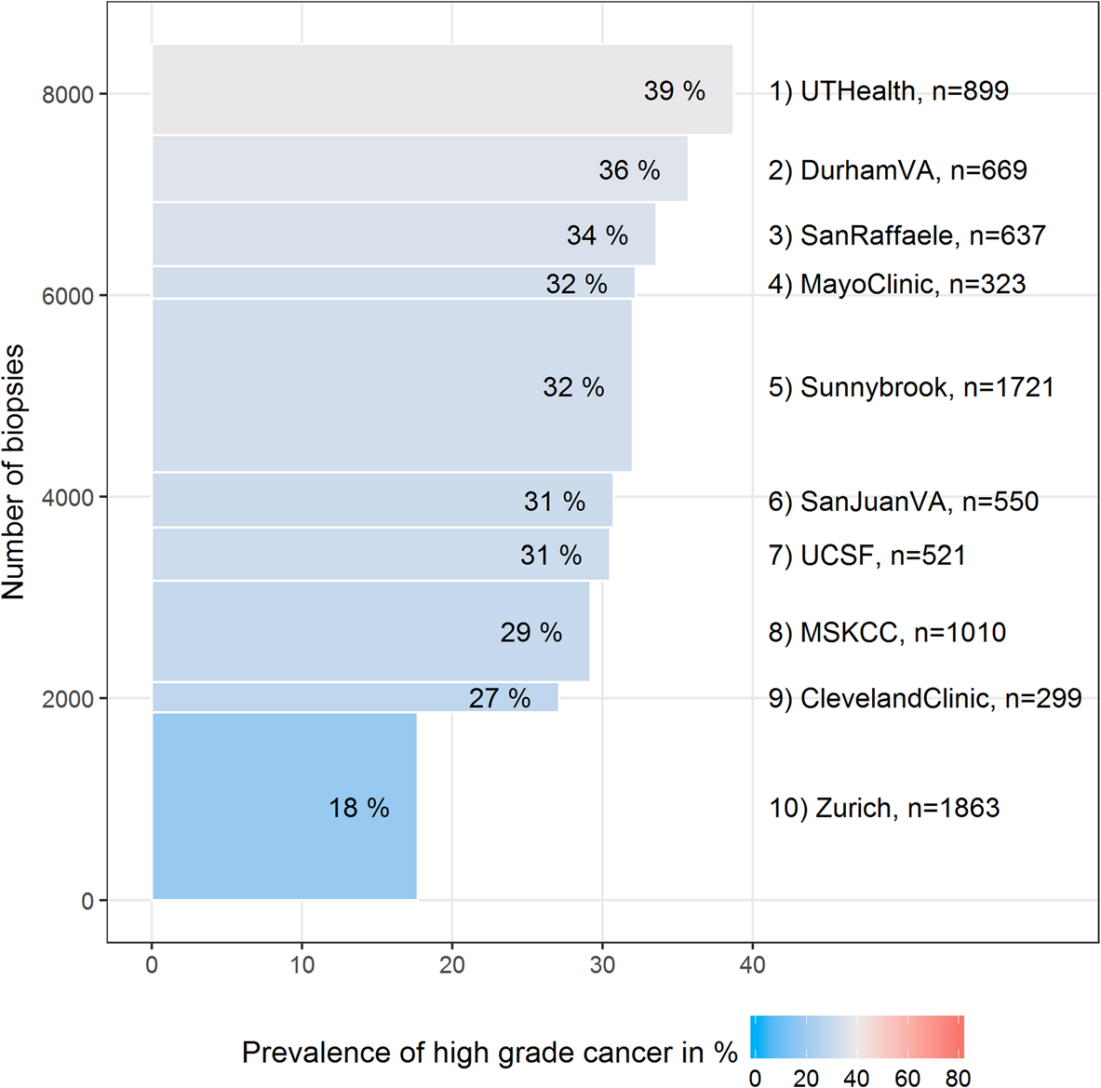
Prevalence of high-grade cancer for the ten PBCG cohorts ordered from highest to lowest along with sample size of the cohort

In total, 8492 biopsies from 8247 patients were available for analysis. Institutional cohorts ranged in size from 299 to 1863, while the prevalence of high-grade prostate cancer ranged from 18 to 39% (Fig. 1).

**Fig. 2:**
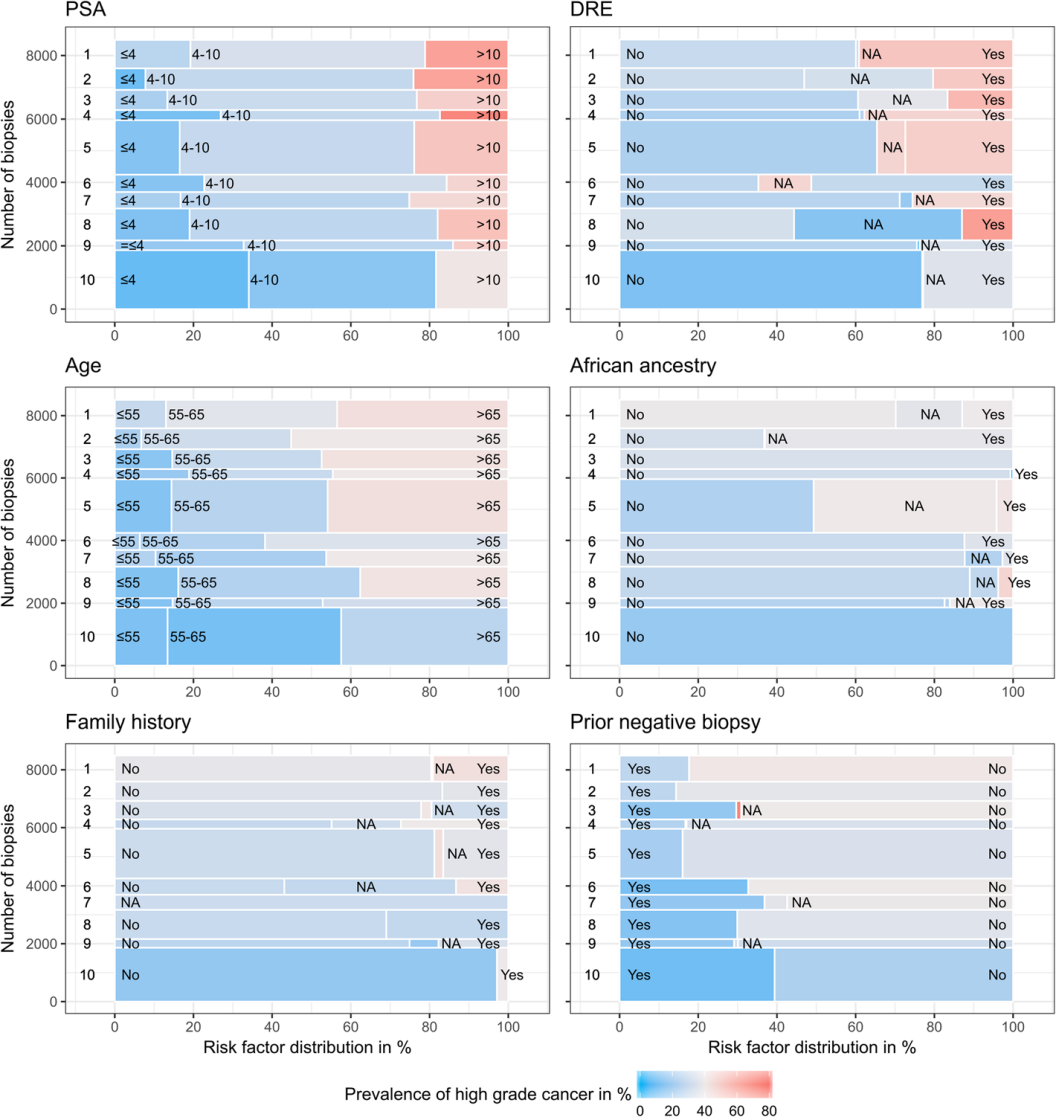
Stacked risk factor distributions on the x-axis and number of biopsies on the y-axis with cohorts ordered from top to bottom by overall prevalence of high-grade cancer as in Fig. 1: 1) UTHealth, 2) DurhamVA, 3) SanRaffaele, 4) MayoClinic, 5) Sunnybrook, 6) SanJuanVA, 7) UCSF, 8) MSKCC, 9) ClevelandClinic, 10) Zurich; NA denotes missing values

To provide insight into sources of the observed heterogeneity in high-grade disease prevalence across cohorts, Fig. 2 maps the cohort-specific prevalence to the cohort-specific risk factor prevalence for each of the six risk factors used in the analysis. For example, for all six risk factors, the large Zurich cohort, which had the lowest prevalence of high-grade cancer, had the highest or second highest proportion of patients with features associated with reduced prostate cancer risk: PSA ≤ 4 ng/ml, normal DRE, age ≤ 65 years, non-African ancestry, negative family history and a prior negative biopsy. In contrast, the two cohorts with highest prevalence of high-grade disease, UT Health and Durham VA, had a higher percentage of patients receiving a biopsy for the first time, and Durham VA had a higher proportion of patients with African ancestry (> 60%) compared to all other cohorts.

**Fig. 3:**
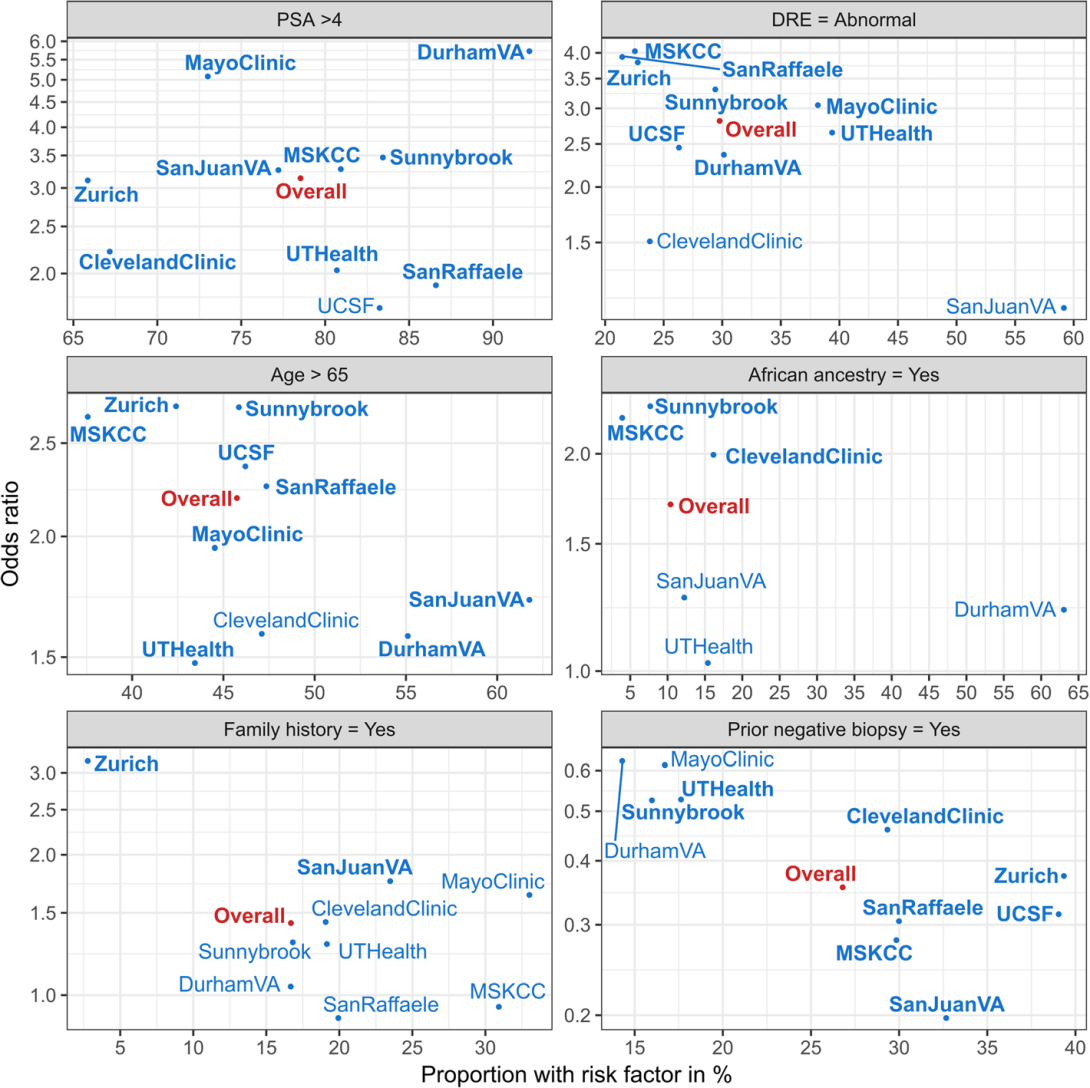
Empirical univariate odds ratios for association between risk factors (age and PSA have been converted to binary factors for the sake of illustration) and high-grade cancer to prevalence of the risk factor in the cohort. Data not shown for African Ancestry for Zurich, San Raffaele, Mayo Clinic and UCSF, and family history for UCSF because numbers were too low to reliably estimate the odds ratios. Bold indicates significance at the 0.05 level; records with unknown risk factors have been excluded

Figure 3 identifies potential outliers in terms of prevalence of risk factors and relationship to odds ratios for high-grade disease. For example, Durham VA had an unusually high percentage of patients of African descent, exceeding 60%, compared to less than 20% in the other cohorts. However, its estimated association with high-grade disease fell in line with that of two other southern US cohorts, San Juan VA and UT Health, both with ORs less than 1.3. The large Zurich cohort had the lowest prevalence of family history (2.8%, compared to over 15% for all other cohorts), but the largest odds ratio for association to high-grade disease (3.2 compared to 1.8 for San Juan VA and less for all others), leveraging the overall estimate towards Zurich (Fig. 3, family history panel).

**Fig. 4:**
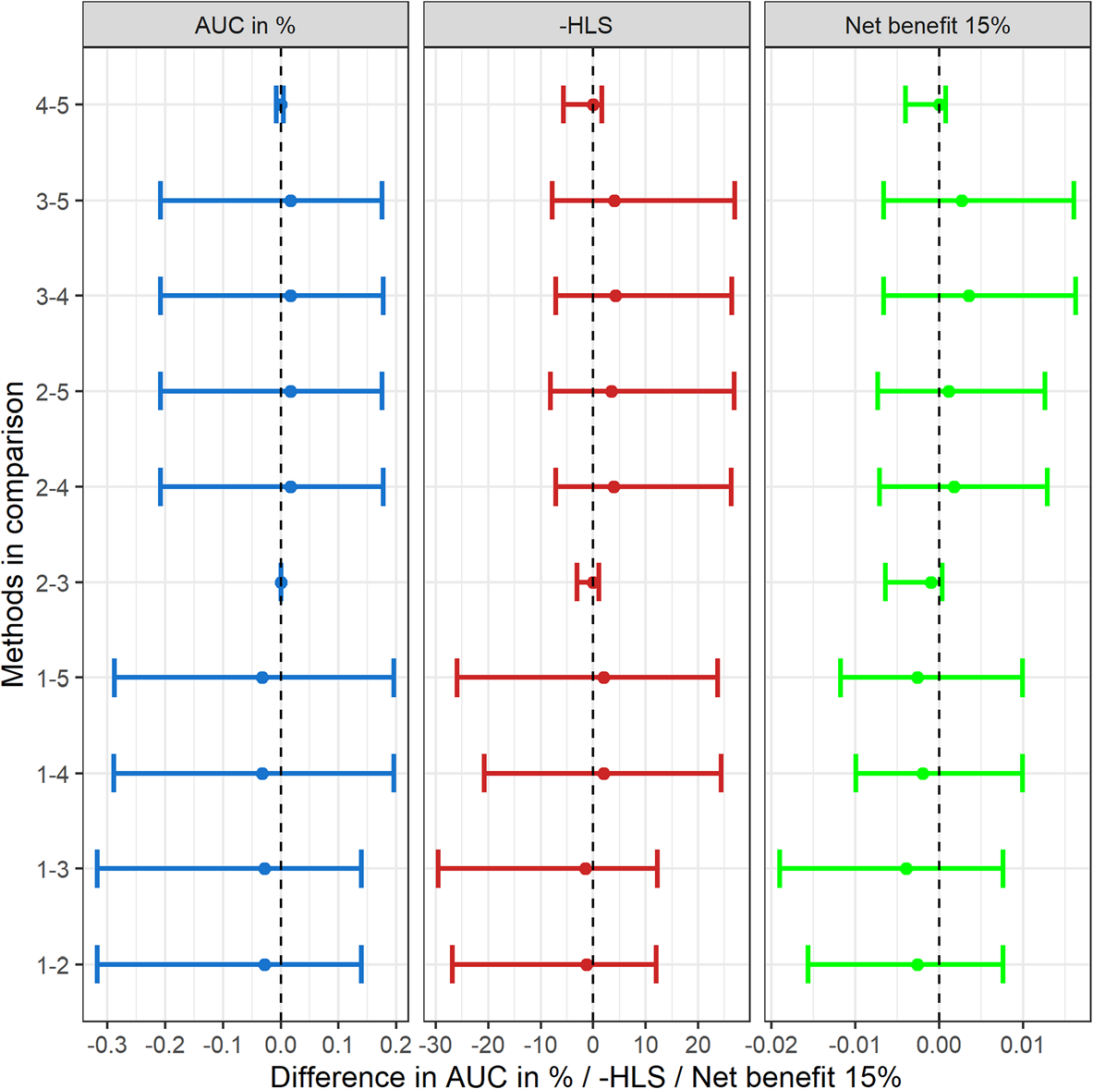
Medians and 95 percentile intervals (2.5 to 97.5 percentile) for comparing the AUC, negative of HLS, and net benefit at the 15% threshold between the five possible prediction methods (numbering according to Table 1: 1-Pooled data, cohort ignored; 2-Pooled data, cohort as random effect, median prediction; 3-Pooled data, cohort as random effect, mean prediction; 4-Meta-analysis, fixed effects by center; 5-Meta-analysis, random effects by center) computed across all 252 choices of five cohorts as test sets with the remaining cohorts as training sets. Positive differences indicate superiority of prediction method listed first for the respective operating characteristic

We compared the five model-based prediction methods of Table 1 across 252 ways to split ten cohorts into five for training and five for testing. The 95% intervals between all pairwise comparisons of two different prediction methods and across all three validation metrics covered zero (Fig. 4). We repeated the cross-validation study leaving out one cohort at a time to see if any of the modeling methods improved upon deletion of a cohort and similarly found no differences (data not shown). Therefore we chose the simplest fixed pooled logistic regression for all further analyses.

**Fig. 5:**
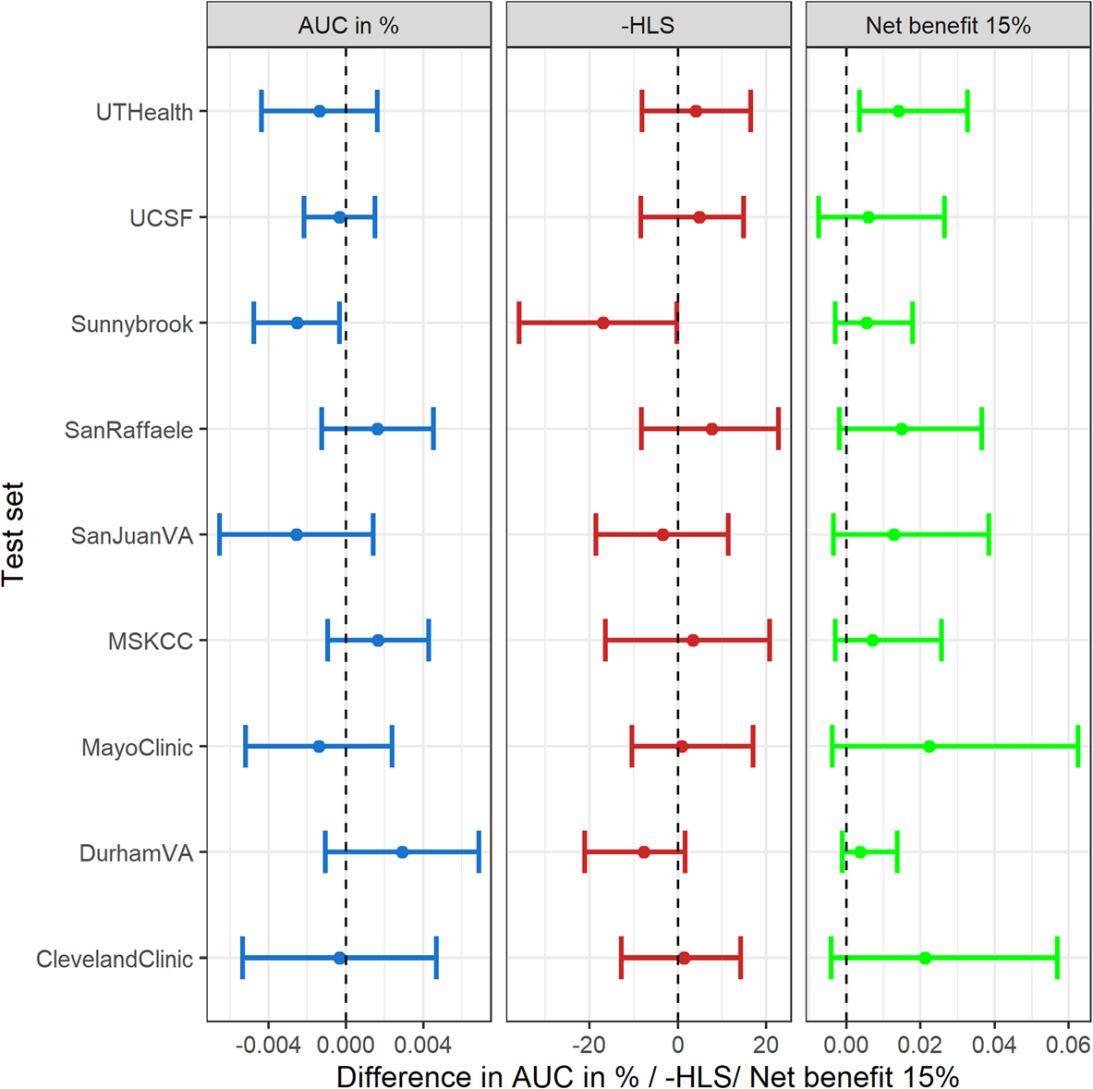
For each PBCG cohort as an individual test set, all other 9 PBCG cohorts were used as a training set to fit a model, which was subsequently evaluated by the AUC, HLS and net benefit at the 15% threshold. The process was then repeated for each test set using the other 8 PBCG cohorts excluding Zurich as a training set. The AUC difference is reported along with 95% confidence intervals. For the negative of HLS and net benefit at 15% threshold, median estimates of difference and 95% percentile intervals (2.5 to 97.5 percentile) are obtained via bootstrapping. Positive values indicate inclusion of Zurich improves the respective performance characteristic for the test set

We investigated the effect of excluding Zurich, the potentially outlying cohort identified by Fig. 3, on predictions for single cohorts when all other cohorts were used in the training set (Fig. 5). Inclusion of Zurich did not significantly reduce the performance characteristics for any of the individual cohorts except for a marginal deterioration of the AUC and –HLS in Sunnybrook, and its inclusion boosted the net benefit in UT Health. We therefore left Zurich in the analysis and used all ten PBCG cohorts for building the final model.

### The global risk prediction model

**Fig. 6:**
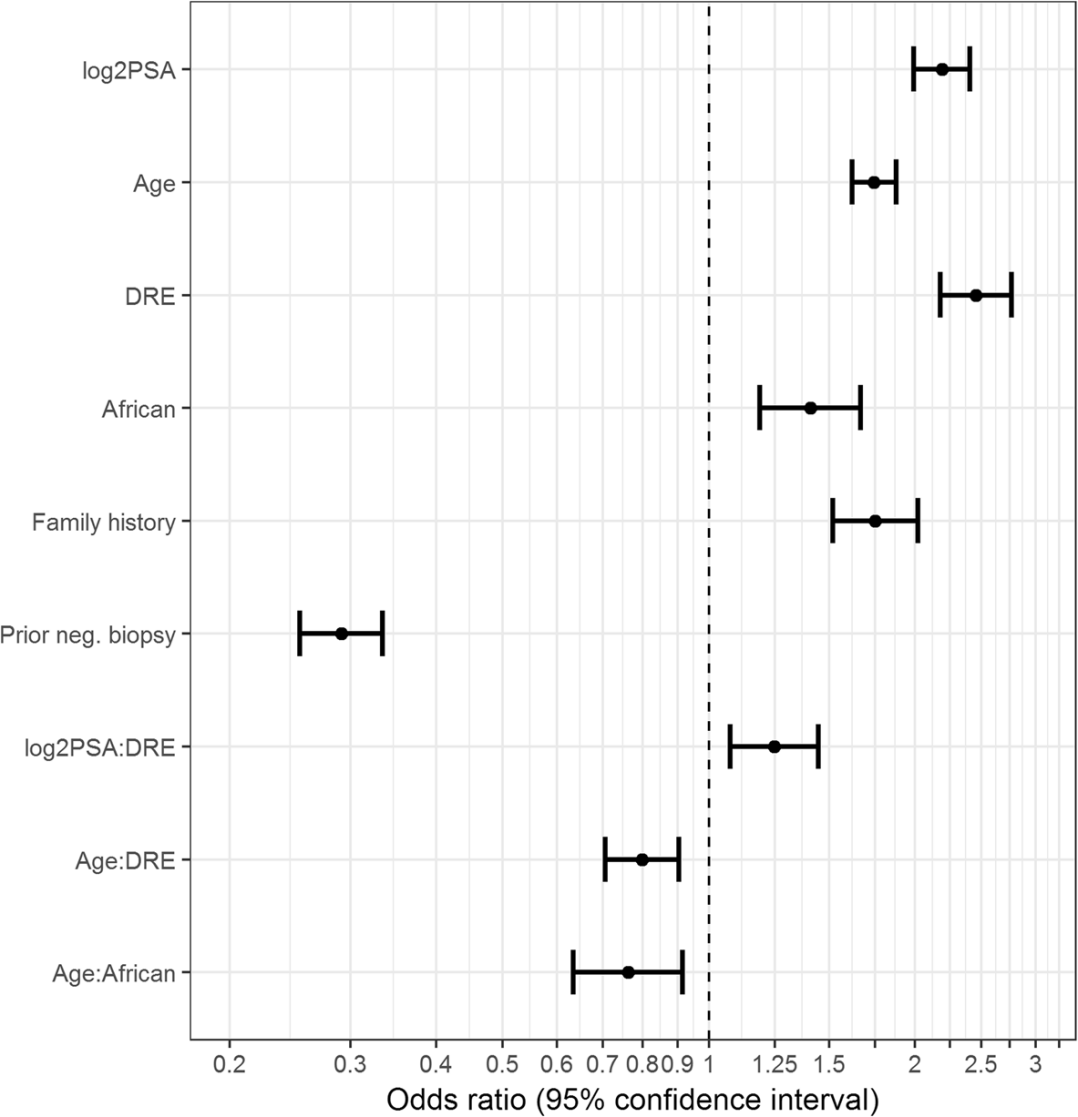
Odds ratios and 95% confidence intervals for the final model fit to the 8492 prostate biopsies from the ten PBCG cohorts. Log2PSA means PSA in ng/ml on the log-base-2 scale, age is in years, DRE is digital rectal exam (0 normal, 1 abnormal), African is African ancestry (1 = yes, 0 = no), Family history is first-degree family history (1 = yes, 0 = no), Prior neg. biopsy is Prior negative biopsy (1 = yes, ever, 0 = never) and colons denote interactions

Figure 6 shows results of the final risk model fit to data from the 8492 prostate biopsies pooled across the 10 cohorts. Higher PSA and age, abnormal DRE, African ancestry and a family history of prostate cancer increased risk of high-grade prostate cancer, while a history of a prior negative prostate biopsy decreased risk. According to our analysis of interaction terms, an abnormal DRE magnified the effect of high PSA on risk, whereas the effect of older age on risk was mitigated in the presence of an abnormal DRE or African ancestry. All *p*-values for odds ratios were less than 0.004.

## Discussion

In the context of our experience with the PBCG, we developed an analytic strategy for developing globally accessible and scalable risk tools. As we are in the modern data science era, we focused first on transparent visualization of center-specific effects to enhance communication among the partners providing data and isolate identifying effects. A center only has the chance to become informed it is an outlier through comparison to other centers, and in some instances such knowledge could improve practice. For example, through the risk factor prevalence versus odds ratio graphs of Fig. 3, the San Juan VA was able visualize its high rate of abnormal DREs (near 60%) compared to other institutions, combined with its low and non-statistically significant association with prostate cancer. In contrast, all other institutions except the Cleveland Clinic had abnormal DRE rates nearly half the size and large significant associations with prostate cancer. The figure also indicated that the outlying San Juan VA effect had no impact on the pooled association, which was later confirmed through the leave-one-cohort-out analyses. We showed univariate associations in Fig. 3, but associations from multivariable models could similarly be displayed.

Internal repeated cross-validation found no differences in performance between five widely used modeling approaches for clustered data: pooling center data while ignoring the effect of center, pooling the data and adjusting for a center effect using random effects with median versus mean prediction, and fixed versus random-effect meta-analysis. All things being equal, the 2-stage meta-analysis methods offer the advantage going forward in that they do not require the transport of data outside of local institutions. Only aggregated study summaries of model coefficients and standard errors are used, thus bypassing the need for ethics board approvals. If local expertise for processing the models is lacking, remote assistance could be provided via R programming scripts on simulated data. Models built on study-summaries are easier to update as well as to scale up for inclusion of more study centers as they do not require re-analysis of the entire data set. So while the current PBCG risk tool is based on pooling the individual-level patient data, going forward the more scalable two-stage meta-analysis method will be used.

As increasingly institutions are capitalizing on and sharing their data, more multi-cohort modeling strategies are being proposed in the literature. Many strategies are possible and the selection of which to adopt should be tightly connected to the goal. Debray et al. focused on the case where individual patient level data were available and where the goal was to calibrate a model to a specific new population, rather than optimizing for global use as the intent here [17]. They proposed a leave-one-cohort-out internal validation because their goal was to optimize validation on specific populations.

Because our goal was to optimize over global populations as those who would access an online tool and to have a balanced sample for testing and training, we selected a 5 cohort train: 5 cohort test set in Fig. 4. This choice is somewhat arbitrary. To investigate the impact on specific cohorts, which can be more interpretable to the data partners, we additionally performed leave-one-cohort out analyses as in Fig. 5. Ahmed et al. published a review of existing individual patient data studies to assess how many incorporated intercept variation, through random effects as considered here or via fixed effects, and Debray et al. recommended guidelines for performing such analyses [19, 20]. They did not compare their models to study-level meta-analysis methods as was performed here.

It seems reasonable to suppose that the more cohorts providing data, the better. However, in a recent online Dream challenge in prostate cancer prediction, the winning team eliminated altogether one of the three large clinical trials provided for training following calibration runs that showed it least matched the held-out test clinical trial used for evaluation [21]. When the goal is to provide a risk tool for ubiquitous use, such as by posting on the internet as for the PBCG model, the intended target is not uniquely defined for calibration purposes. In this case the cohort-level-validation strategy outlined here can be used to determine whether individual cohorts distinguish themselves enough from the rest to warrant elimination.

Despite some strong differences across cohorts in prevalence of the outcome and the risk factor – outcome associations observed in this study, the single cohort exclusion analyses found no cohort outlying to the extent that validation was compromised. If one cohort had substantially differed from the rest, statistical recalibration methods could be applied to tailor the global tool for that population [5, 17]. Recalibration methods are easy to implement as long as individual patient-level data are available from the local institution. Since locally tailored tools can only improve accuracy and hence benefit to the patient, they should be entertained for feasibility and potential impact at any institution that plans to implement a global risk tool for patient counseling.

## Conclusions

We have provided a model-building strategy for developing global risk tools based on multiple heterogeneous cohorts and illustrated it through application to data from a large prostate cancer consortium. We hope the proposed strategy can provide a template for future development of multi-cohort risk prediction tools.

## Data Availability

The datasets used and/or analyzed during the current study are available from the corresponding author on reasonable request.

## Abbreviations

AUC: Area under the receiver operating characteristic curve
DRE: Digital rectal examination
HLS: Hosmer-Lemeshow test statistic
PBCG: Prostate Biopsy Collaborative Group
PSA: Prostate-specific antigen
